# Study of the Photodegradation of PBDEs in Water by UV-LED Technology

**DOI:** 10.3390/molecules26144229

**Published:** 2021-07-12

**Authors:** Meritxell Valentí-Quiroga, Rafael Gonzalez-Olmos, Maria Auset, Jordi Díaz-Ferrero

**Affiliations:** IQS School of Engineering, Universitat Ramon Llull, Via Augusta 390, 08017 Barcelona, Spain; meritxellvalentiq@iqs.url.edu (M.V.-Q.); rafael.gonzalez@iqs.url.edu (R.G.-O.); maria.auset@iqs.url.edu (M.A.)

**Keywords:** flame retardants, polybrominated diphenyl ethers, photodegradation, UV-LED

## Abstract

Polybrominated diphenyl ethers (PBDEs) are persistent organic pollutants that can arrive to water bodies from their use as flame retardants in a wide range of applications, such as electric and electronic devices or textiles. In this study, the photodegradation of PBDEs in water samples when applying UV-LED radiation was studied. Irradiation was applied at three different wavelengths (255 nm, 265 nm and 285 nm) and different exposure times. The best degradation conditions for spiked purified water samples were at 285 nm and 240 min, resulting in degradations between 67% and 86%. The optimized methodology was applied to real water samples from different sources: river, marine, wastewater (effluent and influent of treatment plants) and greywater samples. Real water samples were spiked and exposed to 4 hours of irradiation at 285 nm. Successful photodegradation of PBDEs ranging from 51% to 97% was achieved for all PBDE congeners in the different water samples with the exception of the marine one, in which only a 31% of degradation was achieved.

## 1. Introduction

Flame retardants (FRs) have been used to inhibit ignition and slow down flame propagation celerity, mainly as additives, particularly in electronic devices, adhesives, textiles, plastics and inks [1,2,3,4]. Those compounds are generally organic and halogenated, and especially brominated as in the case of polybrominated diphenyl ethers (PBDEs), hexabromocyclododecane (HBCDs), tetrabromobisphenol A (TBBPA) and polybrominated biphenyls (PBBs).

The PBDE family has been widely used as pyroretardants since the 1970s. They were available in three commercial mixtures [5] containing congeners of different bromination levels: pentaBDE, octaBDE and decaBDE. Despite nowadays production of these mixtures being banned because of their persistence and toxic effects, there is a continued presence of these pollutants in the environment as a result of the historical manipulation and destruction processes of products originally containing the mixtures, with the discharge of wastewater being one of the main liberation sources of PBDEs [6,7,8]. Although the stability of PBDEs is dependent on their individual structure, specifically on their bromination level, the main risk is associated with their high persistence and capacity to bioaccumulate. They are considered to be persistent organic pollutants (POPs) [9]. The susceptibility to bioaccumulate in the tissues of living organisms is one of the main reasons for the growing interest in the analysis of these contaminants in food and in drinking waters.

The distribution of PBDEs throughout the environment is governed by their physicochemical properties, including their hydrophobic behavior (high log K_ow_), low vapor pressure, and high persistence. Moreover, those congeners that present higher bromination levels exhibit lower mobility in comparison to lighter ones, which are less volatile and water soluble. As a consequence, there are scarce bibliographic data available regarding their concentration levels in aquatic environments, as they are found at low concentration rates of pg·L^−1^. According to their lipophilic behavior, they are conventionally monitored through aquatic species and biota rather than being directly measured in water samples because of the difficulty in analyzing such low concentrations [10].

Advanced treatment methods, such as UV treatment, have shown good potential for removing PBDEs from wastewater [11]. Shih et al. [12] studied the photodegradation BDE-209 in water using UV-B light (15W, 300–330 nm), and they observed a complete degradation after 60 min. Agguine et al. [13], for BDE-209 at λ = 228 nm, observed a degradation of 80% after 4 min. Additionally, the removal of PBDE via UV photolysis has been studied in soil matrices [14]. Different UV lamp technologies have been tested to study the photolysis of PBDE, including artificial sunlight lamps, black-light lamps (305–440 nm) and mercury UV lamps (220–400 nm) [14]. However, there is no information on the efficiency of using UV light-emitting diode (UV-LED) technology for PBDE degradation.

Although UV radiation in advanced water treatments has historically been emitted using low- and medium-pressure mercury lamps, they present some intrinsic drawbacks, including their long required start-up times, their low effectiveness, and, primarily, the toxicity caused by the mercury. Thanks to the rapid advance and development in semiconductor technology, UV-LEDs have recently emerged as an alternative to mercury UV lamps. In comparison to mercury UV lamps, UV-LEDs are not only environmentally friendly, avoiding the risk of mercury leaching, but they are also more compact, can be switched off instantly, are more robust, and have relatively longer lifespans [15]. In fact, some UV-LED systems have already been applied to address the problems presented by some emerging environmental contaminants like dye pollutants found in effluent wastewaters from several industries, including the textile and leather, food, and paper sectors [16], and for the disinfection of water [17].

However, one of the main drawbacks of using UV-LEDs in water treatments is that turbidity, caused by the presence of substances such as ions, organic matter, or humic substances, can inhibit the oxidation process by acting as scavengers of radicals and blocking the irradiance of the photoreactors resulting in a decrease of the final yield. Other physicochemical parameters such as temperature or pH might also affect the degradation process [18,19,20].

The present paper assesses the photodegradation of PBDEs in water samples, including real samples (wastewater, marine and superficial), using a UV-LED system and studying different wavelengths (255 nm, 265 nm and 285 nm) and exposure times.

## 2. Results and Discussion

### 2.1. Characterization of the Technical Mixtures

Prior to the photodegradation study, the characterization of each technical mixture was performed to determine their exact composition (congeners and concentration) (Table S1). The most important congeners were BDE-209 (DecaBDE technical mixture), BDE-183 and BDE-153 (OctaBDE technical mixture), and BDE-99 and BDE-47 (PentaBDE technical mixture). These results are in agreement with those found in [5] for equivalent technical mixtures.

### 2.2. Repeatability Study

The variability of the photodegradation process was studied by performing the same experiments on different days (intermediate precision conditions). Replicates from spiked samples (a) non-irradiated, (b) irradiated for 10 min at wavelength 285 nm, and (c) irradiated for 65 min at wavelength 285 nm, were carried out. Mean values of the recovery (%) and relative standard deviation of each congener are presented in Table 1. for each experiment. The recovery is the fraction (%) of the amount spiked for each congener that is in the sample after the irradiation process.

It can be seen from the results (Table 1) that the variability of the analytical procedure with respect to the non-irradiated samples is between 2 and 10% for all of the congeners, including the BDE-209. For BDE-209, the RSD is the highest, since it has the most brominated congener and is the least stable. These are acceptable values according to the standards for the analysis of these contaminants (less than 40% for native congeners) [21].

The results from the experiments under UV-LED radiation (Table 1) correspond to the variability of the own process of irradiation and the degradation produced in the photoreactor. RSD results ranged between 17 and 40% for tetra- to heptabrominated congeners, and around 50% for BDE-209. The variability is clearly higher for irradiated samples than for non-irradiated ones, indicating that photodegradation is not as repeatable as the analytical procedure. It must be taken into account that the PearlBeam photoreactor was originally designed to work with Petri dishes rather than beakers.

### 2.3. Study of Photodegradation Conditions

#### 2.3.1. Irradiation Time

**Figure 1:**
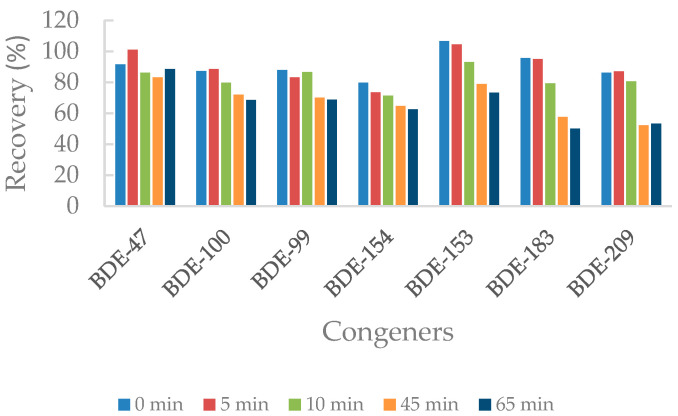
Recoveries (%) of PBDE congeners at different times of irradiation (0 to 65 min) at 255 nm.

The obtained recoveries of the different PBDE congeners of spiked samples after being irradiated at 255 nm at several times are presented below (Figure 1). Despite BDE-138 being present in the PentaBDE technical mixture, this congener is not quantified in the upcoming experiment due to its low concentration level in comparison to the rest of the congeners.

It can be seen in Figure 1 that the longer the time of irradiation is, the higher the degradation of the congeners. Moreover, heavier congeners also experience higher degradation, up to 50% and 47% for BDE-183 and BDE-209. This can be explained on the basis that they present lower stabilities and are more susceptible to debromination processes.

**Figure 2:**
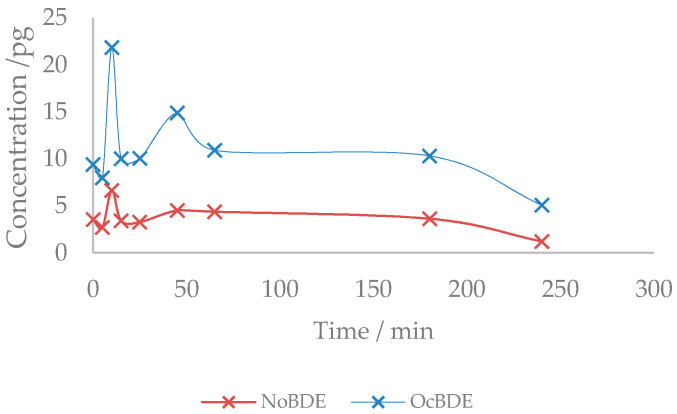
Evolution of octa-BDE and nona-BDE congeners (pg) over time.

At some point, according to the degradation pathways of the congeners, it could be expected that some recoveries are increased as a result of the degradation of some heavier congeners, leading to the formation of lower brominated ones. This effect can be clearly observed in the peak increase of the octa- and nonabrominated diphenyl ethers (octa-BDEs and nona-BDEs) (Figure 2), which are produced as a direct debromination of BDE-209, while BDE-209 itself exhibits a reduction in concentration, from an initial amount of approximately 9 ng to a final amount of 2 ng.

**Figure 3:**
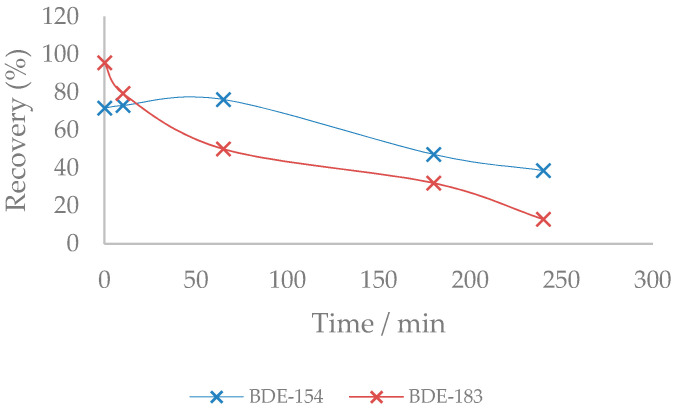
Recoveries (%) of BDE-183 and BDE-154 congeners over time.

The same tendency of higher brominated congeners to suffer direct debromination is exhibited by BDE-183, leading to BDE-153 and BDE-154 congeners, which, in the end, both lead to BDE-28. This can be seen especially in the obtained recoveries of BDE-154, which undergoes a slight increase in its recovery at around 65 min of irradiation, while BDE-183 decreases continuously (Figure 3). These degradation pathways are also described in the degradation of BDE-209 [22].

**Figure 4:**
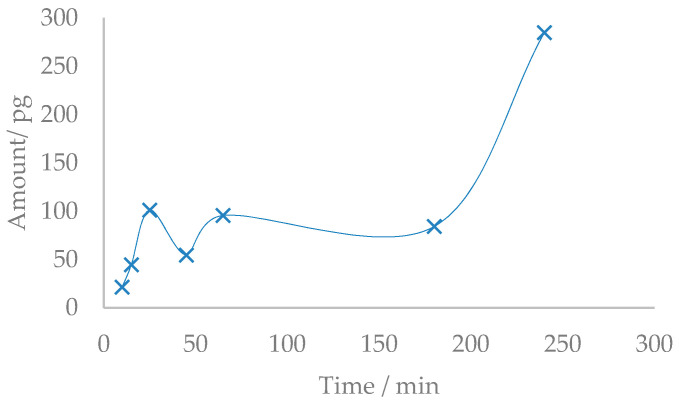
Evolution of BDE-28 (pg) over time.

However, this direct debromination pathway is less clear when focusing on the recoveries of lower brominated congeners such as BDE-99 and BDE-100, which in both cases exhibit only 19% degradation, and BDE-47, which was only degraded by 9%. These results might be the consequence of the degradation of higher congeners, which at the same time increase the concentration of the lower brominated ones. This debromination phenomenon can be clearly observed with the emergence and rise of BDE-28 (Figure 4).

Despite BDE-28 not originally being present in the technical mixtures, it was detected in the analysis of the samples subjected to degradation in the photoreactor. The quantification (Figure 4) of this congener was performed by applying the response factor of native standards from the calibration curve. The increase of BDE-28 in the first part of the graphic can be explained as a result of the degradation of other congeners with a higher bromination degree. The decrease of BDE-28 at around min 50 could be due to the degradation of BDE-28 itself, whereas the formation from other congeners is low. The increase in the last part of the graphic can be explained by the degradation of a lot of highly brominated congeners yielding BDE-28 at the end. In fact, the appearance of some other peaks can be qualitatively observed in the chromatograms at different times. These are new PBDE congeners that are formed as a consequence of the degradation process.

On the basis of these observations it can be stated that debromination is one of the photodegradation pathways, although it may not be the only one. It would be expected that some hydroxylated derivatives might be formed during the process as a result of the ionization of water and the interaction with the PBDEs [23,24].

#### 2.3.2. Irradiation Wavelength

**Figure 5:**
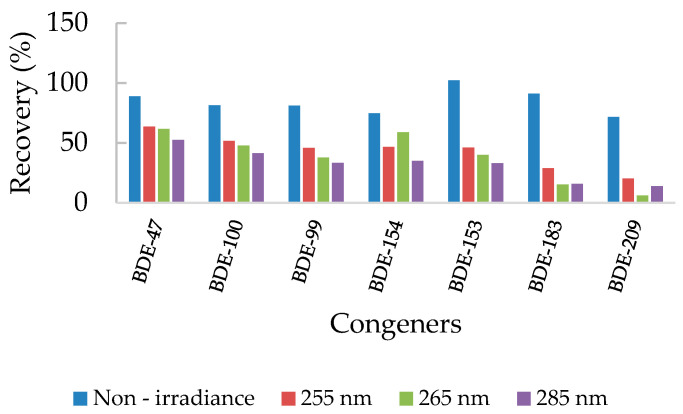
Recoveries of PBDE congeners after 4 hours of irradiation at different wavelengths.

As the reactor can work at three different wavelengths (255 nm, 265 nm, and 285 nm), the photodegradation at these other wavelengths was also tested with spiked water samples, irradiating them for 4 hours. Higher degradation was achieved when irradiating at 285 nm for 4 h in comparison to 265 nm and 255 nm (Figure 5).

The differences between the results are not enough to determine whether the improvements in degradation are a consequence of the applied wavelength or are the result of the variability in their own processes. Moreover, it should also be considered that irradiance in LED systems varies depending on the wavelength to prevent overheating. The average irradiance values were 4.21 × 10^−3^ mW/cm^2^, 2.90 × 10^−2^ mW/cm^2^ and 7.03 × 10^−2^ mW/cm^2^ for 255, 265 and 285 nm, respectively. Thus, it can be seen that more energetic wavelengths have lower irradiance, which could help to explain the obtained results. However, since for most congeners the best degradation is achieved when irradiating at 285 nm, reaching a degradation of 67–86% for all congeners, compared to 255 nm (36−80%) and 265 nm (58−94%), further experiments with real samples were performed at this wavelength.

Direct photolysis of PBDEs depends on the absorption spectra of the congeners. Although spectra might vary from one to another congener according to the number and position of bromines, all of them absorb in the range of 280 nm to 400 nm being their molar absorptivity much bigger in the UV-B range (280−315 nm) than in the UV-A (315−400 nm). Moreover, congeners with higher bromine substitution present maximum absorbance at higher wavelengths [24]. All the studied congeners present a maximum peak of absorbance around 285 nm [24,25], which could explain the better degradation results at this wavelength. Finally, the best conditions for studying the degradation of PBDE congeners in real water samples were determined to be 4 h of irradiation time at 285 nm.

### 2.4. Photodegradation Study in Real Samples

#### 2.4.1. PBDE Concentrations in the Samples

As mentioned above, before studying the degradation process of PBDEs in real water samples, a first analysis was performed to determine the intrinsic concentrations of the analytes in each sample (Table 2). The total concentration of PBDEs was calculated as the sum of all the congeners, assuming the limit of detection (LOD) to be the maximum concentration for those that were below the limits (upperbound). To evaluate whether the analyzed samples met the specified environmental quality standards (EQS) in the current legislation in the Directive 2008/105/EC, the sum of the congeners BDE-28, -47, -100, -99, -154 and -153 was also calculated.

As shown in Table 2, all the samples presented some PBDE congeners. While BDE-28 was not detected in any of the samples, BDE-209 was detected in all of them, presenting the highest concentrations in samples from wastewater treatment plants especially in the influent of the wastewater treatment plant (WWTP) from Sta. Maria de Palautordera at a concentration of 188 pg·g^−1^.

According to the total amount of PBDEs, the marine and the river samples presented the lowest concentrations (0.3 pg·g^−1^ and 0.2 pg·g^−1^ respectively). The congener concentration was similar for most congeners but the concentration of BDE-209 in the marine sample was near twice than in the river sample. The greywater sample presented higher concentrations of the sum of PBDEs in comparison to the marine and river sample (1.9 pg·g^−1^), but lower than those from the WWTPs. The major contribution in the greywater to the final PBDE concentration was BDE-209 (1.62 pg·g^−1^). Apart from this congener, BDE-99 and BDE-47 were also detected in similar concentrations compared to the previously discussed samples. Congeners BDE-154 and BDE-153 represented the lowest contribution at concentrations of 0.007 pg·g^−1^ and 0.004 pg·g^−1^ respectively. As expected, effluent samples from the WWTPs presented lower concentrations of pollutants in comparison to their respective influent levels. Both effluents from WWTPs presented similar concentrations of total PBDEs of 3.4 pg·g^−1^ (Sta. M. Palautordera) and 2.3 pg·g^−1^ (St. Celoni). Higher concentrations of PBDEs were detected in influent samples, where BDE-209 played a major role in the contribution to the total PBDE concentration. Sample from the WWTP in St. Celoni presented a concentration of 9.9 pg·g^−1^ whereas the sample from the plant in Sta. M. de Palautordera presented a concentration of 191 pg·g^−1^. If excluding BDE-209, both influents of the WWTPs presented similar profiles of BDE congeners. However, the sample from Sta. M. de Palautordera presented higher concentrations compared to the sample from St. Celoni. Fewer congeners were detected in both of the WWTPs effluent samples in comparison to their relative influents. Only BDE-47 and BDE-209 were detected in the effluent from St. Celoni WWTP, and neither BDE-154 nor BDE-153 were detected in the effluent from Sta. M. de Palautordera.

PBDE concentrations in water samples reported in literature are summarized in Table 3. Due to the intrinsic properties of PBDEs, it is not easy to find reported concentrations of these pollutants in water samples. Moreover, not always the same PBDE congeners are studied and reported, which also makes more difficult to compare the results.

Given these data (Table 3), a comparison of the obtained results from the analyzed samples can be made. PBDE levels of the marine sample (0.1 pg·g^−1^ excluding BDE-209 and 0.241 pg·g^−1^ for BDE-209) were similar to reported levels in marine waters of Japan and Barcelona. The total amount of PBDEs with the exclusion of BDE-209 (0.1 pg·g^−1^) of the analyzed sample from river Onyar was lower than reported concentration of Guadalquivir, and slightly higher than the one in river Seine. However, it was similar to the reported values of other European rivers. Concentration of BDE-209 in this sample matched with the reported levels in the Seine and European rivers. Concentration of PBDEs in the analyzed greywater sample was, considering the sum of all congeners, lower in comparison to the other reported levels of greywaters from Sweden and Spain. Levels of PBDE in influents from both WWTP were lower than reported ones from plants in Australia, China and Korea. However, the high concentration of BDE-209 in the influent from Sta. M. de Palautordera was similar to the reported levels in China and Korea. Concentrations of congeners in both effluents were similar to the published values in WWTPs from Australia and Shanghai.

#### 2.4.2. Degradation of PBDE Congeners

In view of the obtained results, (Table 2) as PBDE concentrations of individual congeners were in most cases below 0.10 pg·g^−1^, fortification of samples was performed before studying the degradation process in the photoreactor to achieve a final concentration of 200 pg·g^−1^ of all the congeners. However, as the influent sample from the WWTP in Sta. Maria de Palautordera presented the highest concentration of BDE-209 (188 pg·g^−1^), fortification was excluded in this case. Once the samples were spiked, irradiation at 285 nm was applied for 4 h. Then, samples were analyzed to quantify PBDE congeners and determine the loss by degradation (Table 4).

As can be seen in Table 4, degradation of congeners was successfully experienced in all samples except in the marine one, where only a range from 14% to 36% of degradation was achieved. In the other samples, degradation ranging from 75% to 99% was obtained for all the congeners with the exclusion of the influent from the WWTP in Santa Maria de Palautordera, where slightly less degradation (55−65%) was observed for BDE-47, BDE-100, BDE-99 and BDE-209. However, neither BDE-153 nor BDE-154 were detected, and some formation of BDE-183 was produced (124%) in this sample.

With the exception of the influent sample from Santa Maria de Palautordera, the heavier the congener, the higher the degradation. This does not necessarily imply that higher congeners are more easily degraded than lighter ones, but that degradation of heavier congeners led to the formation of lower brominated congeners, and the concentration of the latter can be increased. According to the literature, the most common degradation pathways with respect to photodegradation mechanisms are reductive debromination and intermolecular elimination of HBr to produce lower brominated PBDEs, brominated dibenzofurans (PBDFs) and other derivatives depending on the media [34]. Reductive debromination mechanisms depend on both position and number of bromine atoms in the congeners, with the atoms in ortho and meta positions being more susceptible to debromination than para positions [35]. Moreover, the composition of the matrix including suspended and dissolved solids and other species like humic substances, metal cations or halide anions, as well as solvent effects, might influence the degradation kinetics. However, indirect photolysis might also be experienced and promoted by the presence of photosensitizers such as organic matter and ferric ions, which produce reactive organic substances (ROS) such as ^1^O_2_, ^·^OH, or O_2_^−^, which enhance degradation pathways. Although heavier PBDE congeners, like BDE-209, are mainly degraded through direct reductive debromination, indirect photolysis has a significant role in the degradation of lighter congeners [34,36]. This could justify the better degradation results obtained in real water samples in comparison to the results with spiked purified water. The presence of organic matter and other components in real matrices, which could previously lead to thinking they would negatively interfere with the degradation process by hindering it and lowering final yield, actually increases the degradation processes. Thus, under the effects of the UV radiation the plausible formation of ROS (different from the hydroxyl) promoted degradation reactions.

Nevertheless, according to the results obtained from the marine sample, it seems that high concentrations of salt (mainly sodium chloride) do not produce the same beneficial effect. In this case, the drawbacks could be a consequence of the similar behavior of chlorine and bromine which stunts PBDE degradation.

**Figure 6:**
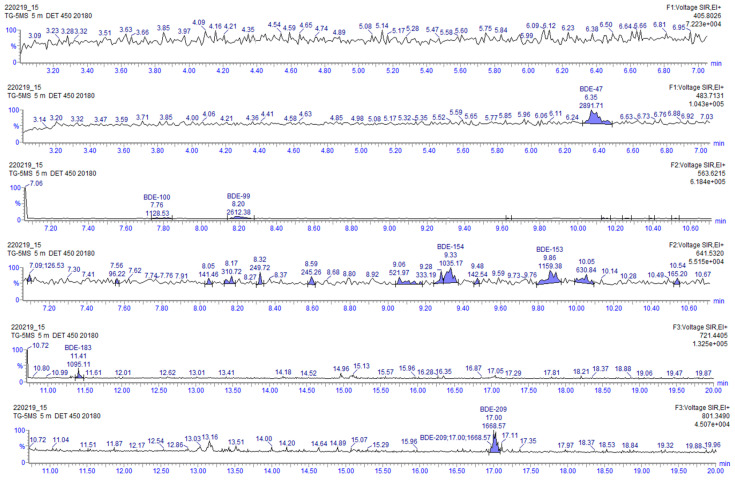
HRGC-HRMS chromatogram of the PBDE congeners in the river sample before photodegradation.

**Figure 7:**
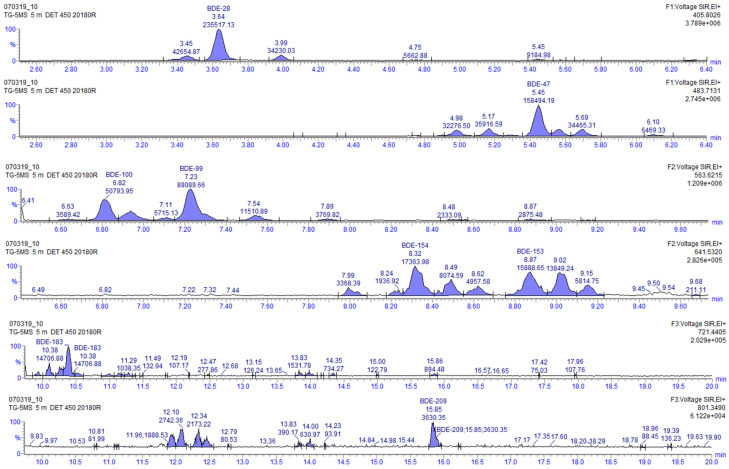
HRGC-HRMS chromatogram of the PBDE congeners in the river sample after photodegradation.

Additional information about the degradation process can be obtained by analyzing the chromatograms before and after the UV-LED treatment. In the chromatogram after the UV-LED treatment it can be observed that new peaks are formed, which must be degradation products (Figure 6 and Figure 7).

These products can be clearly observed, especially close to BDE-28, BDE-47, BDE-99, BDE-100, BDE-154 and BDE-153 (Figure 7). When analyzing the sample extract by gas chromatography with electron capture detector (GC-ECD), the same peaks can be identified, and no other different peaks can be detected. This implies that all those new products that are derived from PBDEs must also be other PBDE congeners, since they are monitored at the same masses. Some specific degradation pathways for PBDE congeners that have been previously reported in organic solvents could help in understanding the increasing concentration and formation of lower brominated congeners. In particular, the degradation of BDE-153, which directly decomposes into BDE-101, BDE-99 and BDE-118 [37], or BDE-47, which has been reported to directly decompose into BDE-28 and BDE-17, leading to BDE-15, BDE-8, BDE-4, BDE-3 and BDE1 [37,38,39]. Moreover, quantification of these peaks was performed by applying response factor of the closest native congeners from the calibration curve. This allowed us to compare the initial concentration of the sum of all the native PBDE congeners with the concentration of the newly appearing congeners after the degradation. The result was that at the end of the whole process, approximately 1.5% of new congeners were formed, in contrast to the 87% of the degradation observed in the experiment. In view of all these obtained results, and in agreement with the published literature about the degradation pathways of BDE-209 [22,23,24], a possible identification of the appearing products detected in the chromatograms of the analyzed samples was performed according to their retention times and elution order (Table 5).

From all the compounds listed in Table 5, only seven congeners were originally detected in the analyzed water samples (BDE-209, BDE-183, BDE-154, BDE-153, BDE-100 and BDE-47) before the degradation experiments. However, after the irradiation, some new congeners were detected. Those products can be clearly observed close to the original PBDE congeners. Debromination depends on the number and position of bromine and can be affected by matrix interactions with the solvents. Wang et al. [38] reported differences in the degradation yields of PBDE-47 in isooctane and nonane. The lack of oxygen/OH radicals in nonane limited the formation of phenols due to the minor oxidation reactions in comparison to isooctane where the yield of brominated phenols reached 10 times higher concentrations. Fang et al. [37] compared the degradation rates of some PBDE congeners in which the slowest photolysis congener was BDE-100 instead of the lower brominated congener BDE-28, indicating that bromine substitution pattern also has implications in photodegradation rates. Similar results were reported by Eriksson et al. when comparing pure methanol, methanol/water, and THF, where pure methanol solvent acted as a better hydrogen donor, increasing quantum yields [40]. Moreover, previously described degradation pathways suggest effects of structural conformations in which loss of bromine atoms is more likely to occur in ortho or meta positions in comparison to in para position [22,23,24,37,38,41], which could make it possible to understand degradation pathways in terms of debromination. However, it must be considered that photodegradation using UV-LED technology not only involves direct debromination mechanisms, it also implies the formation of some PBDE derivatives. Especially when working in aqueous media, hydroxylated BDEs (OH-BDEs) or chlorinated BDEs can be formed as a reaction product of the hydroxyl radicals/chlorine with the BDE congeners. Further works will study the possible formation of these other groups of compounds in water matrices.

## 3. Materials and Methods

### 3.1. Reagents and Standards

Silica 60 (70–230 mesh) and sodium sulphate were acquired from Merk (Darmstadt, Germany), hexane and nonane was supplied by Honeywell (Charlotte, NC, USA), and sulphuric acid was acquired from Scharlau (Barcelona, Spain). PBDE standards and 13C labeled solutions were from Cambridge Isotope Laboratories Inc. (New Haven, USA) and PBDE technical mixtures (pentabrominated, octabrominated and decabrominated) were supplied by LGC Standards (Teddington, UK).

### 3.2. Photoreactor Set Up

**Figure 8:**
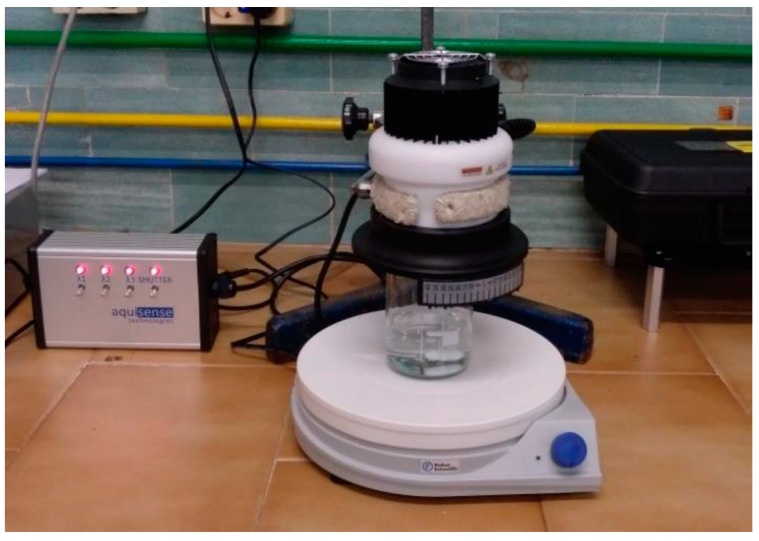
PearlBeam T255/265/285 device.

Irradiation of the samples was performed using a PearlBeam T255/265/285 device (Figure 8) manufactured by Aquisense Technologies (Erlanger, USA). The instrument consisted of a UVinaire™: a lamp module containing small, state-of-the-art UV-C LEDs with up to three selectable wavelengths (255 nm, 265 nm and 285 nm), a collimating tube and a stand to easily place the sample. Although this device was originally designed to perform experiments with Petri dishes, experiments were performed using 200 mL beakers placed right under the lamp (Figure 8). It is important to consider that as the distance from the light source to the sample must have a direct influence in the irradiance of the sample, the distance between the beaker containing the sample and the lamp was kept constant. Moreover, continuous stirring of the samples was performed to achieve homogenous irradiation of the whole sample. Stirring was set at the same output to ensure the same vortex effect in all samples. By fixing these variables, controlled conditions for the experimentation were achieved. The incident fluence rates of the UV-LEDs device were measured using a radiometer (ILT 2400) with a detector (SED 270, typically designed for UV-LEDs with emission spectral range of 215–355 nm) supplied by International Light Technologies and calibrated using a conventional potassium ferrioxalate actinometer. The measured irradiance incident to the surface of the treated sample (i.e., incident irradiance) was calculated by 10 mm horizontal measurement intervals across the entire sample plane aperture, with the sensor head matching the height of the water surface. The values measured by the radiometer represent the integrated fluence rates under the emission spectra of the UV-LEDs.

### 3.3. Characterization of the Technical Mixtures

For the characterization of the technical mixtures, the commercial mixtures of penta-BDE, octa-BDE and deca-BDE were dissolved in nonane and, after the addition of the injection standard 13C BDE-139, they were instrumentally analyzed by gas chromatography coupled to high-resolution mass spectrometry (GC-HRMS) (see Section 3.6.2).

### 3.4. Study of Photodegradation Conditions

Several exposure times, ranging from 5 min to 240 min, as well as different irradiation wavelengths were tested with spiked water samples in order to establish the best degradation conditions. First of all, samples were irradiated at 255 nm at 5 min, 10 min, 15 min, 25 min, 45 min, 65 min, 180 min and 240 min. Then, in view of the obtained results, samples were exposed 240 min at the different available wavelengths: 255 nm, 265 nm and 285 nm. In both cases, non-irradiated samples were also analyzed as control. Moreover, a repeatability study was performed to determine the variability of the whole process by performing the same experiments in different days (intermediate precision conditions). Replicates from non-irradiated and irradiated samples at 285 nm under 65 min of irradiation were performed four times, and 10 min samples were performed three times.

### 3.5. Samples

#### 3.5.1. Spiked Samples

To establish the working conditions to achieve the best degradation results, the first trials were performed with spiked samples of purified (Elix-Essential 10) water. A combined standard of 200 ng·mL^−1^, containing penta-BDE, octa-BDE and deca-BDE technical mixtures, was prepared to spike the samples. This mix was quantified against individual PBDE congeners to know which congeners were present and at what concentration. The characterization of individual congeners in each technical mixture was performed against individual PBDE standards. Therefore, the exact concentration of each spiked PBDE congener coming from the different technical mixtures was calculated. Samples (100 mL were spiked with 375 μL of this combined standard (characterized mix of technical mixtures) to achieve a concentration of 300 ng·mL^−1^ in the final extract.

#### 3.5.2. Real Samples

**Figure 9:**
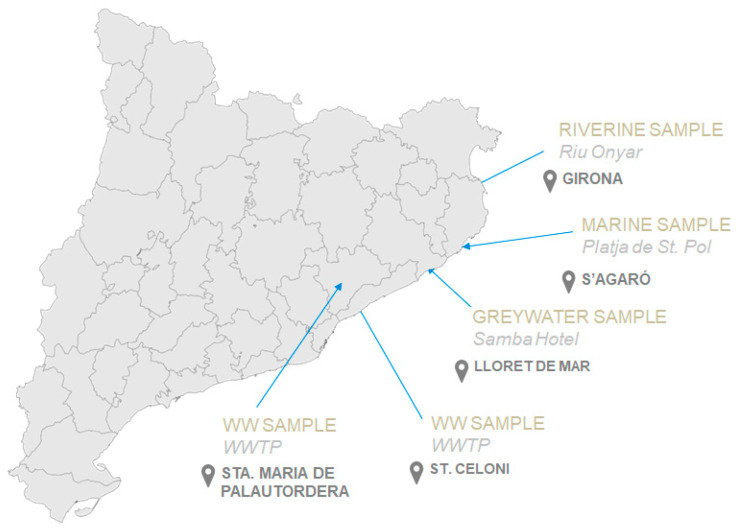
Map of samples and sources.

Once those optimum degradation conditions in spiked purified water were established, photo-degradation of PBDEs with UV-LED was tested in real water samples. The study of the photo-degradation in real water samples makes it possible to determine some matrix effects that might interfere in the process, such as the presence of organic matter or salts. Samples from five different sources were selected to perform the analysis in order to cover a diversified scenario. All of them were collected in the Catalonia area (Figure 9) in glass sampling bottles, previously rinsed with acetone and air-dried, and stored under refrigeration at 4–6 °C after sampling. A superficial continental water sample was collected in the river Onyar, in its flow in the center of Girona city (41.977570, 2.823815). At the beach of Sant Pol in S’Agaró (Sant Feliu de Guíxols, 41.789783, 3.047935) a marine sample was collected. Wastewater samples coming from the influent and the effluent of the WWTPs of Sant Celoni and Santa Maria de Palautordera were also provided. Finally, a greywater sample was supplied by Hotel Samba from Lloret de Mar (Girona).

Physical differences between samples can be easily observed at a glance. Samples from the influent of the WWTP treatment plant presented higher turbidity and suspended matter and a yellowish coloration. The greywater sample also presented high turbidity, but in this case with a whitish coloration, which was a consequence of the presence of surfactants and soaps. Samples from both WWTP effluents and the river presented similarities in terms of appearance, all being practically transparent. However, they presented a slight coloration, denoting the presence of dissolved or colloidal matter. The marine sample was, at first sight, the one that presented the least turbidity and coloration. Before testing photo-degradation of PBDEs in real water samples in the reactor, each sample was analyzed to determine its intrinsic concentration of PBDEs. As PBDE concentrations of individual congeners were in most cases below 0.10 pg·g^−1^, fortification of samples was performed to study the degradation process in the photoreactor. However, as the influent sample from the WWTP in Sta. Maria de Palautordera presented the highest concentration of BDE-209, fortification was not considered in this case. According to the concentration levels and obtained results in the previous degradation studies with spiked Elix water, fortification was performed to achieve a final concentration of 200 ng·mL^−1^ in the final extract of 25 μL.

### 3.6. Sample Analysis

#### 3.6.1. Extraction and Clean-up

The samples were spiked with a mixture of ^13^C labeled extraction standards. Then, they were extracted with hexane (approximately 10% of the total sample volume) three times. All the collected organic fractions were then passed through an anhydrous sulphate column (35 g) and eluted with 40 mL of hexane to remove remaining water. Extracts from real water samples were additionally purified through acidic silica columns. Final extracts were concentrated under reduced pressure up to 5 mL and then adjusted to final volume of 25 μL for the GC-HRMS analysis with a smooth nitrogen flow. Nonane was added as a keeper in this step. Finally, 25 μL of the injection standard 13C BDE-139 (200 ng mL^−1^) was added.

#### 3.6.2. Instrumental Analysis

Analyses of the purified extracts were performed by GC-HRMS with an Agilent HP 6890-N chromatograph coupled to an AUTOSPEC ULTIMA Mass Spectrometer from Waters. Chromatographic conditions are summarized in Table S2. HRMS operated in EI ionisation (35 eV) at a resolving power of 10,000. Acquisition was performed in SIM mode in different time windows. The monitored masses of PBDEs and the monitoring times are presented in Table S3. A calibration curve containing a mix of the congeners was injected in order to determine response factors. The standards’ concentrations were in the range 1 ng mL^−1^ to 2500 ng·mL^−1^ except for decaBDE, which was ten times more concentrated in all the standards. Quantitation in the samples was carried out by the isotope dilution method. The total concentration of PBDEs was calculated as the sum of all the congeners assuming the concentration of LOD for those that were below the limits (upperbound). To evaluate whether the analyzed samples met the specified environmental quality standards (EQS) set in the European Directive 2008/105/EC [42], the sum of congeners BDE-28, -47, -100, -99, -154 and -153 was also calculated.

## 4. Conclusions

Successful degradation of PBDE congeners was achieved with UV-LED light, ranging from 51% to 97% for all the PBDE congeners in the different water samples, except for the marine one, in which only 31% degradation was achieved. Better degradation results were obtained with real water samples in comparison to spiked purified samples. This could be explained by the presence of other substances, such as organic matter and other ions that might react and enhance the whole processes. According to the obtained chromatograms of the samples after irradiation, debromination might be one of the degradation paths for PBDE congeners when exposed to UV-LED light due to the formation of new PBDE congeners. However, despite the formation of different PBDE congeners during the degradation process, it was calculated that degradation yield was still higher (87%) in comparison to the formation of new congeners (1.5%). Finally, new research lines could be suggested to deeply understand how degradation of PBDEs under UV-light exposure undergoes regarding degradation rates and specific pathways in water matrices. Other complementary analytical techniques and instruments could be employed in order to determine specific degradation products and establish the most feasible mechanisms. Moreover, an individual study of the congeners would also be interesting to complement and point out all those degradation pathways, as well as some studies regarding the toxicity of the effluents after the photodegradation process.

## Figures and Tables

**Table 1:** Degradation mean values, expressed as recoveries (%) and RSD (%) of the replicates for the variability study.

Congener	No Irradiance (*n* = 4)		10 Minutes of Irradiation (*n* = 3)		65 Minutes of Irradiation (*n* = 4)	
	Mean (%)	RSD (%)	Mean (%)	RSD (%)	Mean (%)	RSD (%)
BDE-47	89	3	73	35	74	18
BDE-100	83	5	65	35	59	17
BDE-99	84	4	66	40	54	22
BDE-154	72	7	60	36	58	27
BDE-153	99	8	77	33	60	30
BDE-183	96	2	69	29	40	34
BDE-209	77	10	53	51	35	49

**Table 2:** Concentrations (pg·g^−1^) of PBDE congeners in real water samples.

Congener	Marine Sample	Superficial Continental Sample (River)	Influent WWTP Sta. M. de Palautordera	Effluent WWTP Sta. M. de Palautordera	Influent WWTP St. Celoni	Effluent WWTP St. Celoni	Greywater Sample
	Concentration/pg·g^−1^	Concentration/pg·g^−1^	Concentration/pg·g^−1^	Concentration/pg·g^−1^	Concentration/pg·g^−1^	Concentration/pg·g^−1^	Concentration/pg·g^−1^
BDE-28	<0.010	<0.012	<0.193	<0.193	<2.004	<1.201	<0.116
BDE-47	0.013	0.013	1.316	0.154	0.831	0.244	0.018
BDE-100	0.007	<0.006	0.193	0.016	0.104	<0.014	<0.017
BDE-99	0.015	0.015	0.729	0.051	0.307	<0.085	0.044
BDE-154	0.013	<0.012	0.076	<0.036	0.051	<0.057	0.004
BDE-153	0.017	<0.021	0.106	<0.079	0.055	<0.127	0.007
BDE-183	0.022	0.019	<0.128	<0.059	0.079	<0.093	<0.034
BDE-209	0.241	0.124	188	2.828	6.483	0.507	1.624
TOTAL	0.3	0.2	191	3.4	9.9	2.3	1.9
TOTAL according to EQS	0.1	0.1	2.6	0.5	3.4	1.7	0.2

**Table 3:** Bibliographic data summary for PBDE concentration in different water samples.

Sample	Region	Congeners	Concentration/pg·g^−1^	Reference
Marine water	Japan	ΣBDE-28,-47,-99,-100,-153,-154,-183	0.11	[26]
		BDE-209	0.19	
Marine water	Spain (Barcelona coastline)	ΣBDE-28,-47,-99,-100,-153,-154,-183	0.6	[27]
		BDE-209	<LOD	
Riverine water	France (Seine)	ΣBDE-28,-47,-99,-100,-153,-154,-183	0.002–0.004	[28]
		BDE-209	0.20	
Riverine water	Spain (Guadalquivir)	ΣBDE-28,-47,-99,-100,-153,-154,-183	0.7	[27]
		BDE-209	<LOD	
Greywater	Sweden	BDE-209	11–330	[29]
Greywater	Spain	ΣBDE-28,-47,-99,-100,-153,-154,-183	0.7	[27]
		BDE-209	8.56	
WWTP influent	Australia	ΣBDE-28,-47,-99,-100,-153,-154,-183	40.29	[30]
		BDE-209	60	
WWTP effluent		ΣBDE-28,-47,-99,-100,-153,-154,-183	7.04	
		BDE-209	2	
WWTP influent	China (Harbin)	ΣBDE-28,-47,-99,-100,-153,-154,-183	1.56	[31]
		BDE-209	150	
WWTP effluent		ΣBDE-28,-47,-99,-100,-153,-154,-183	0.31	
		BDE-209	15.7	
WWTP influent	Korea	ΣBDE (11 congeners)	69.6−183	[32]
WWTP effluent		ΣBDE (11 congeners)	1.59−2.34	
WWTP influent	China (Shanghai)	ΣBDE (19 congeners)	4.64	[33]
WWTP influent		ΣBDE (19 congeners)	2.55	

**Table 4:** Degradation (%) of PBDE congeners in real water samples after being irradiated 4 h at 285 nm.

Congener	Marine Sample	Continental Sample	Influent WWTP Sta. M. Palautordera	Effluent WWTP Sta. M. Palautordera	Influent WWTP St. Celoni	Effluent WWTP St. Celoni	Greywater Sample
BDE-47	34	81	57	75	90	86	89
BDE-100	29	84	55	82	90	89	92
BDE-99	31	91	65	89	95	94	96
BDE-154	37	87	-	83	91	90	92
BDE-153	14	91	-	88	94	95	94
BDE-183	21	97	-24	97	97	98	97
BDE-209	36	99	51	98	94	99	99
TOTAL	32	94	51	93	94	96	97

**Table 5:** Identification of the degradation intermediates and PBDE congeners.

Name	Number	Retention Time/min
2,4′,6-TrBDE	BDE-32	3.46
2,4,4′-TrBDE	BDE-28	3.65
TetraBDE	Tetra –a (non-identified)	3.99
2,4,4′,6-TeBDE	BDE-75	4.98
2,2′,4,5′-TeBDE	BDE-49	5.17
2,2′,4,4′-TeBDE	BDE-47	5.45
2,4,4′,5-TeBDE	BDE-74	5.56
2,3′,4,4′-TeBDE/ 2,2′,3,4′-TeBDE	BDE-66/BDE-42	5.69
3,3′,4,4′-TeBDE	BDE-77	6.10
2,2′,4,5,6′-PeBDE	BDE-102	6.65
2,2′,4,4′,6-PeBDE	BDE-100	6.82
2,2′,4,5,5′-PeBDE	BDE-101	6.94
2,3′,4,4′,6-PeBDE/ 2,3′,4,5,5′-PeBDE	BDE-119/BDE-120	7.11
2,2′,4,4′,5-PeBDE	BDE-99	7.23
2,2′,3′,4,5-PeBDE/ 2,3′,4,4′,5-PeBDE	BDE-97/BDE-118	7.54
2,2′,3,4,4′-PeBDE	BDE-85	8.00
3,3′,4,4′,5-PeBDE/ 2,2′,4,4′,6,6′-HxBDE	BDE-126/ BDE-155	8.23
2,2′,4,4′,5′,6-HxBDE	BDE-154	8.34
2,2′,3,4,5′,6-HxBDE	BDE-144	8.49
HexaBDE	Hexa-a (non-identified)	8.62
2,2′,4,4′,5,5′-HxBDE	BDE-153	8.90
2,2′,3,4,4′,6-HxBDE	BDE-139	9.04
2,2′,3,4,4′,6′-HxBDE	BDE-140	9.16
2,2′,3,4,4′,5′-HxBDE	BDE-138	9.53
2,3,4,4′,5,6-HxBDE	BDE-166	9.96
2,3,3′,4,4′,5-HxBDE	BDE-156	10.10
2,2′,3,4,4′,6,6′-HpBDE/ 2,2′,3,3′,4,4′-HxBDE	BDE-184/128	10.30
2,2′,3,4,4′,5′,6-HpBDE	BDE-183	10.40
2,2′,3,4,4′,5,5′,6-OcBDE	BDE-203	12.09
2,2′,3,3′,4,5,5′,6,6′-NoBDE	BDE-208	12.35
2,2′,3,3′,4,4′,5,6,6′-NoBDE	BDE-207	12.46
2,2′,3,3′,4,4′,5,5′,6-NoBDE	BDE-206	13.99
DeBDE	BDE-209	15.87

## Data Availability

The data presented in this study are available in present article and supplementary material.

## Supplementary Materials

The following are available online, Table S1: Concentration (ng mL^−1^) of detected congeners in technical mixtures, Table S2: GC-HRMS conditions for the analysis of PBDEs, Table S3: Monitored masses and monitoring windows for PBDE analysis by GC-HRMS.

## Sample Availability

Samples of the compounds are not available from the authors since they are solutions that are not easily transported.
