# Supplementary material for: Study of the Photodegradation of PBDEs in Water by UV-LED Technology
Source: Molecules. 2021 Jul 12;26(14):4229. doi: 10.3390/molecules26144229 (PMC8303597; doi:10.3390/molecules26144229)
Supplement: Supplementary file 1 [file molecules-26-04229-s001.zip › molecules-1254639-supplementary.pdf]

Supplementary information

Table S1: Concentration (ng ml-1) of detected congeners in technical mixtures.

Technical mixture	Congener	Rt /min	Concentration / ng·ml^{-1}
PentaBDE	BDE-47	12.081	57.13
	BDE-100	13.498	17.59
	BDE-99	13.907	79.47
	BDE-85	14.598	4.07
	BDE-154	15.019	6.80
	BDE-153	15.561	6.86
	BDE-138	16.251	1.31
	BDE-183	17.095	0.80
OctaBDE	BDE-154	15.020	2.58
	BDE-153	15.562	16.23
	BDE-183	17.096	100.34
	BDE-209	22.970	2.19
DecaBDE	BDE-209	22.970	197.14

Table S2: GC-HRMS conditions for the analysis of PBDEs.

Column	TG – 5MS
Length, column diameter and phase thickness	5 m x 0,25 mm x 0,25 µm
Carrier gas	He
Flow	1 ml·min^{-1}
Temperature of the injector	270 ºC
Split flow	Splitless 1 min
Injection volume	2 µl
Temperature program	120 ºC (3 min) at 10ºC/min up to 300 ºC (7 min)
Transfer line temperature	320 ºC

Table S3: Monitored masses and monitoring windows for PBDE analysis by GC-HRMS.

Time range /min	Homologous	^{12}C monitored masses		^{13}C monitored masses	
0 – 6.75	TriBDE	405.8046	407.8006	417.8429	419.8409
	TetraBDE	483.7131	485.7111	495.7533	497.7513
6.75 – 9.73	PentaBDE	563.6215	565.6195	575.6618	577.6598
	HexaBDE	641.5320	643.5300	653.5723	655.5703
9.73 – 20	HeptaBDE	721.4405	723.4385	733.4808	735.4788
	OctaBDE	799.3511	801.3491	811.3914	813.3893
	NonaBDE	879.2596	881.2575	891.2998	893.2978
	DecaBDE (-2Br)	797.2354	799.3334	809.3756	811.3736
